# Prenatal carrier screening for spinal muscular atrophy among pregnant Thai women

**DOI:** 10.3389/fmed.2025.1566417

**Published:** 2025-06-23

**Authors:** Chayada Tangshewinsirikul, Panyu Panburana, Maneerat Prakobpanich, Takol Chareonsirisuthigul, Donniphat Dejsuphong, Thipwimol Tim-Aroon, Chaiyos Khongkhatithum, Thanyachai Sura, Atchara Tunteeratum, Duangrurdee Wattanasirichaigoon

**Affiliations:** ^1^Division of Maternal-Fetal Medicine, Department of Obstetrics and Gynaecology, Faculty of Medicine Ramathibodi Hospital, Mahidol University, Bangkok, Thailand; ^2^Department of Obstetrics and Gynaecology, Faculty of Medicine Ramathibodi Hospital, Mahidol University, Bangkok, Thailand; ^3^Department of Pathology, Faculty of Medicine Ramathibodi Hospital, Mahidol University, Bangkok, Thailand; ^4^Section for Translational Medicine, Faculty of Medicine Ramathibodi Hospital, Mahidol University, Bangkok, Thailand; ^5^Division of Medical Genetics, Department of Pediatrics, Faculty of Medicine Ramathibodi Hospital, Mahidol University, Bangkok, Thailand; ^6^Division of Neurology, Department of Pediatrics, Faculty of Medicine Ramathibodi Hospital, Mahidol University, Bangkok, Thailand; ^7^Division of Medical Genetics, Department of Internal Medicine, Faculty of Medicine Ramathibodi Hospital, Mahidol University, Bangkok, Thailand

**Keywords:** spinal muscular atrophy, SMA, prenatal, carrier screening, attitude

## Abstract

**Objectives:**

To investigate the acceptance rate for spinal muscular atrophy (SMA) carrier screening among Thai pregnant women, their attitudes toward the prenatal screening, carrier rate, and the frequencies of *SMN2* copy numbers.

**Methods:**

Singleton pregnant women who aged ≥ 18 years, with a gestational age of ≤ 14 weeks at their first visit, were invited to participate the study. All participants completed the questionnaire: Section I—demographic data. Then, they received a pre-test group counseling, followed by an offer of SMA carrier testing at no cost and completion of the questionnaire: Section II—awareness and attitudes toward the screening and Section III—reasons for their choosing “to have” or “not to have” the screening done. Only those having the test done and undergoing post-test counseling were asked to complete the questionnaire: Section IV—attitudes toward the screening process.

**Results:**

The preexisting knowledge about SMA was low (30.8%). After pre-test counseling, the majority of participants (94.4%) realized the severity of SMA and its burden to the families, leading to a high acceptance rate for carrier screening at 91.4% (181/198). Most participants (92.4%) agreed to have a fetal diagnosis if they were found to be a couple at risk. The genetic screening revealed a SMA carrier rate of 2.2% (1 in 45), and a high frequency of ≤ 2 copies of *SMN2* (98.3%). Four participants were found to be carriers, but none of their partners were carriers, yielding no couples at risk in this study. After disclosure of the screening test result, all the participants expressed a feeling of gladness that they had had the test done. The majority (98%) suggested that SMA carrier screening should be offered to all pregnant women and that the cost of testing should be covered by the government and/or by their health coverage schemes (95.5%).

**Conclusion:**

The high acceptance rate and positive attitude toward prenatal SMA carrier was demonstrated among Thai pregnant women. Data from the present study highlight urgent needs for endorsement from professional society and public health policy in advancing the SMA carrier screening program in Thailand.

## Introduction

Spinal muscular atrophy (SMA) is a severe neuromuscular disorder that results in progressive muscle weakness, with an estimated incidence of 1 in 6,000–10,000 births worldwide (1). SMA is divided into four clinical types depending on the age of onset and clinical progression. Type I SMA (Werdnig–Hoffmann) has a clinical onset within the first three months of life, usually leading to respiratory failure and death by two years of age if left untreated; however, the life expectancy and quality of life of many patients have been much improved with adequate respiratory and nutritional management. The onset of type II SMA is 6–18 months of age, and the affected children may sit independently but cannot stand or walk, with 70% surviving beyond 25 years of age. Patients with type III SMA (Kugelberg–Welander), with the onset at ≥ 18 months, have difficulty climbing stairs and running and often have a normal lifespan. Type IV SMA, or adult-onset SMA, is described as a slow progression of muscle weakness beginning at around 30 years of age and with no reduction in life expectancy (2). Type I SMA has the highest incidence among SMA, at 50%–60% (2–5). However, there is a marked difference between the incidence and prevalence of type I SMA because of its low survival (6, 7). Among the living individuals with SMA, types II and III together account for 75%–83% (6–9).

Survival motor neuron 1 (*SMN1*) gene, located on 5q11.2–q13.3, is the disease-causing gene of SMA. Approximately 95%–98% of SMA cases are caused by a homozygous deletion of exon 7 or exons 7–8 of the *SMN1* gene, with 3%–5% being due to point mutation or compound heterozygosity between the deletion and point mutations (2). *SMN2* is a highly homologous gene of *SMN1*, they are located next to each other. The complete absence of *SMN2* yields no clinical effect on healthy individuals. The copy number of *SMN2* rather modifies the severity of SMA (2, 6, 10). Most people have two copies of *SMN1*, each on the homologous chromosome, and two copies of *SMN2*, each on the homologous chromosome as well (2–11). The higher number of *SMN2* copy number is associated with less severity of the SMA phenotypes (1 copy of SMN2: 96%–type I SMA; 2 copies: 79%–type I; 3 copies: 54%–type II and 31%–type III/IV; ≥ 4 copies: 88%–type III/IV) (2). Therefore, simultaneous copy number detection of *SMN1*-exon 7 (or exons 7–8) and *SMN2*-exon7 is widely used for molecular diagnosis and prognostication for SMA (2, 12). As for carrier screening in the general population, copy number analysis of the *SMN1* is a preferred method owing to its high throughput, rapid turnaround time, and economical reason (13–15).

Carrier frequency of SMA in the general population is high at approximately 1 in 33–94 worldwide (2, 11, 13, 15, 16), and potentially with the most severe phenotype due to the high frequency of ≤ 2 copies of *SMN2* among the carriers (15, 17–21). Screening for SMA carriers before conception or early in pregnancy, regardless of religion or ethnicity, has been recommended by the American College of Medical Genetics (2008) and by the American College of Obstetricians and Gynecology (2017) (22, 23).

Thailand is a middle-income country with well-established universal health coverage (UHC) since 2002^1^ Almost all Thai citizens are currently covered by one of the three healthcare coverage schemes: 74% under the Thai UHC, 7% are covered by the Civil Servant Medical Benefit Scheme (CSMBS, for government/state employees), and 19% under the Social Security Scheme (SSS for working adults). Notably, 93% of Thai children are covered by the UHC. Prenatal screening for thalassemia (carrier rate 40%) has been offered as the national policy since 1992, with a good coverage of 98% of pregnant women (24, 25). Carrier frequency of SMA among the Thai population is 1 in 40 (26, 27), but SMA carrier screening is not included in the national program or routine antenatal care in the country. Newborn screening for SMA is not offered, and specific treatments for SMA (such as mRNA-modifying treatment and gene therapy) are not reimbursable under the existing healthcare benefit schemes in the country. Carrier testing for SMA and reproductive choices are offered to only families with previously affected children/relatives with SMA.

To explore the potential for establishing carrier screening for SMA during the prenatal period, we set off to conduct this study. Our objectives were to assess the acceptance rate of the carrier screening among pregnant Thai women, to evaluate their attitudes toward the screening, and to determine the prevalence of SMA carriers and the frequencies of *SMN2* copy numbers.

## Materials and methods

### Study design and participants

This was a prospective and descriptive study conducted between 1 March 2021 and 31 March 2022 at the antenatal care clinic at Ramathibodi Hospital. The protocol was approved by the Ramathibodi Hospital Institutional Review Board (COA, MURA2020/1420) and complied with the Declaration of Helsinki. The study was registered as a clinical trial (registration number NCT04859179).

Eligible participants were women with singleton pregnancy, who were aged ≥ 18 years and capable of reading and understanding Thai, and with a gestational age of ≤ 14 weeks at their first visit with our antenatal care clinic. The reasons for setting the upper time interval of GA 14 weeks in this study were that the turnaround time of genetic testing was 2 weeks, and genetic counseling and testing of the partner could take place at 16–18 weeks GA. Prenatal diagnosis would be performed at 18–20 weeks, if the couple was found to be an at-risk couple, which allows for a definite fetal diagnosis at 20–22 weeks. Thailand’s legal gestational age limit for medical abortion is 24 weeks. We approached the pregnant women and their accompanying partners, provided them with information about this project, and invited them to participate in the study. However, only the women were asked to complete the questionnaire and represent the sum decision/answer of the couple. This is because, at the end of the day, we need one unified decision/answer from each couple. For those who agreed to participate, a written informed consent was obtained.

Exclusion criteria were refusal to give written consent, withdrawal during or after the completion of the study, and insufficient data obtained from the questionnaire.

### Questionnaire

A Thai-language questionnaire was developed using information from a literature review and then validated by six clinical experts: two maternal and fetal medicine specialists, two pediatric geneticists, and two clinical scientists. The reliability of the questionnaire: Sections II and IV were 0.741 and 0.731, respectively, as assessed by using Cronbach’s alpha. The actual questionnaire was translated into English and provided as supporting information (Supplementary Appendix 1).

The questionnaire contained four sections, I–IV. Section I focused on the participants’ demographic data, including age, education, occupation, religion, gravida, and previous history of fetal anomalies. Section II asked about attitudes toward the severity and burden of SMA and prenatal SMA carrier screening. This section comprised nine question items, including the awareness of the severity of SMA and its burden on the patients and families, the usefulness and interest in having prenatal carrier screening for SMA and their confidence in the reliability of the test offered, agreement in having a definitive test for fetal diagnosis, possible considering termination of pregnancy as an alternative if the fetus to be found affected, opinion on having SMA carrier screening as part of standard antenatal care, and their willingness to self-pay for the screening test. The answer options of Section II were “agree,” “not sure,” and “disagree,” (Supplementary Appendix 1).

As for Section III, we explored the reason(s) and/or rationale thinking of the participants for choosing “to have” or “not to have” the carrier screening test. Multiple choice answers were provided to choose, and the participants could choose more than one answer as wanted. We also asked the participants’ opinions on a reasonable price for the screening test if it had to be self-pay, and who (individuals, organizations, health schemes, or else), in their opinion, should be responsible for the cost of testing if the screening would become part of standard antenatal care in the country.

Section IV consisting of five question items; was about the participants’ attitudes and reflections on their immediate past experience with going through the SMA carrier screening process including pre-test and post-test counseling: overall feeling/satisfaction with the screening process, the usefulness of the information provided, the essence of post-test counseling, the whole process is time worthy, and that the carrier screening should be offered to all pregnant women during the antenatal visit. The answer options for each question were “agree,” “not sure,” and “disagree” (Supplementary Appendix 1).

### Data collection

All the participants, after the completion of Section I of the questionnaire, were provided with pre-test group counseling delivered by trained maternal and fetal medicine specialists using a PowerPoint presentation which lasted for about 10 min. The participants then received the Thai-language brochure to read in a designated area; this was to allow the participants time to digest the information received and self-check their understanding. The slide presentation for the group counseling and the brochure contained similar information, including the incidence, clinical presentations, natural course and outcomes of SMA; treatment options and the efficacy of available treatments, affordability of the treatment in the country; carrier rate and carrier screening; and prenatal diagnosis and its downstream services. Of note, the Thai-language brochure was approved by a maternal and fetal medicine specialist (CT), two pediatric geneticists (TT and DW), and a pediatric neurologist (CK). The Thai-language brochure used in this study and its English translation are provided as supporting information (Supplementary Appendices 2, 3).

**FIGURE 1:**
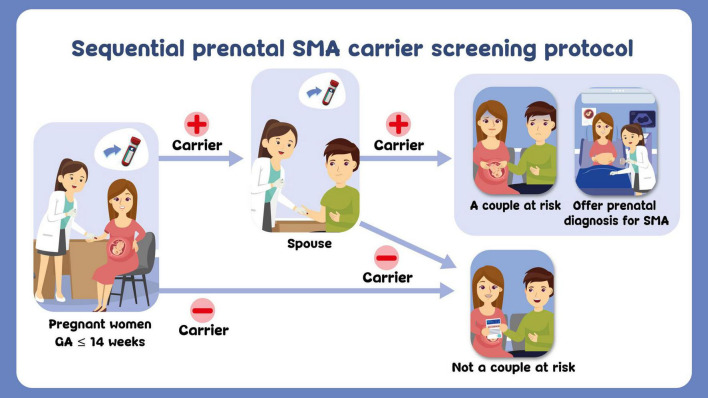
Sequential prenatal SMA carrier screening protocol.

After pre-test counseling, a free-of-charge SMA carrier testing (as part of this research) was offered to all participants and they were asked to complete the questionnaire Sections II and III, regardless of their choosing to have the test done or not. A sequential prenatal SMA carrier screening protocol was established to use in this research (Figure 1). For those choosing not to have the test done, the study ended at this point. About those choosing to have carrier testing performed, 5-ml of venous blood sample was taken for *SMN1* and *SMN2* analysis.

Post-test counseling for each individual was offered by a maternal and fetal medicine specialist at an antenatal care clinic, upon the result became available. Then, the participants were requested to finalize Section IV of the questionnaire. For those who were found to be a carrier, their partner was counseled and offered carrier testing (with no charge). Prenatal diagnosis would be discussed with a couple at risk.

### Carrier testing and copy numbers analysis for SMN1 and SMN2

To detect the copy number of SMN1 and SMN2, we performed multiple ligation-dependent probe amplification (MLPA) analyses, using a commercially available MLPA kit for SMA (SALSA P021), following the manufacturer’s protocol (MRC Holland, Amsterdam, the Netherlands). The MLPA reactions included internal quality controls and negative controls. The PCR products were analyzed using the Applied Biosystems 3500 Genetic Analyzer (Thermofisher Scientific, MA, USA) and Coffalyser.net software (MRC Holland), following the manufacturer’s instructions. The tests were performed at the Human Genetics Laboratory, Ramathibodi Hospital. Carrier status, in the present study, is defined as having only 1 copy of SMN1 exon 7. The results of carrier testing were reported to the clinician researchers (CT, PP, and MP) within two weeks.

### Data and statistical analyses

Categorical variables were described as the number (percent). Parametric continuous variables were expressed as a mean and standard deviation. Chi-square and *t*-tests were used for statistical analysis, employing STATA software package version 16.1 (Stata Corp., College Station, Texas, USA). The answer options of Sections II and IV: “agree,” “not sure,” and “disagree,” were then analyzed on a Likert scale with scores of 3, 2, and 1, respectively.

## Results

There were 237 pregnant women who attended our antenatal clinic during the time of the study; 35 did not meet the inclusion criteria (27 having GA > 14 weeks, and 8 having twin pregnancies), leaving 202 eligible participants. Only 4 out of 202 declined to sign the consent, yielding 198 individuals who took part in the study. The mean age of the participants was 32 ± 5.1 years and over half (66.2%) of them had a Bachelor’s degree or higher. None had a child or family relative(s) with SMA. Two-thirds of the participants said that they had never heard of SMA before (Table 1). About 60% of the participants were accompanied by their partners to the antenatal clinic, and around 39% came alone.

**TABLE 1:** Demographic data of participants.

Characteristics	Total participants *N* = 198 (%)	Accepted screening test *N* = 181 (%)	Declined screening test *N* = 17 (%)	*p*-value^a^
Maternal age (years; mean ± SD)	32 ± 5.1	32 ± 5.1	33.6 ± 5	0.182
Place of current residence				1.000
Bangkok	152 (76.8)	139 (76.8)	13 (76.5)	
Other provinces	46 (23.2)	42 (23.2)	4 (23.5)	
Religion				0.285
Buddhist	176 (88.9)	162 (89.5)	14 (82.4)	
Christian	5 (2.5)	5 (2.8)	0	
Muslim	17 (8.6)	14 (7.7)	3 (17.6)	
Highest education				0.473
Below bachelor’s degree	67 (33.8)	59 (32.6)	8 (47.1)	
Bachelor’s degree	113 (57.1)	105 (58)	8 (47.1)	
Above bachelor’s degree	18 (9.1)	17 (9.4)	1 (5.9)	
Occupation				0.160
Company/private sector employee	68 (34.3)	63 (34.8)	5 (29.4)	
Causal worker/day laborer	6 (3)	5 (2.8)	1 (5.9)	
Housewife/unemployed	22 (11.1)	21 (11.6)	1 (5.9)	
Personal business/self-employed	21 (10.6)	16 (8.8)	5 (29.4)	
Government/state enterprise employee	38 (19.2)	37 (20.4)	1 (5.9)	
Healthcare professional	30 (15.2)	27 (14.9)	3 (17.6)	
Other	13 (6.6)	12 (6.6)	1 (5.9)	
Family’s average monthly income				0.677
< 15,000 Baht	19 (9.6)	17 (9.4)	2 (11.8)	
15,000–29,999 Baht	104 (52.5)	94 (51.9)	10 (58.8)	
30,000–50,000 Baht	56 (28.3)	53 (29.3)	3 (17.6)	
> 50,000 Baht	19 (9.6)	17 (9.4)	2 (11.8)	
Type of healthcare coverage				0.397
Universal health coverage (UHC)	8 (4.1)	7 (3.9)	1 (5.9)	
Social Security Scheme (SSS)	81 (40.9)	74 (40.9)	7 (41.2)	
CSMBS	47 (23.7)	41 (22.7)	6 (35.3)	
Cash	62 (31.3)	59 (32.6)	3 (17.6)	
Previous child with a genetic disorder/congenital disabilities/anomaly				1.000
Yes	8 (4)	7 (3.9)	1 (5.9)	
No	188 (94.9)	172 (95)	16 (94.1)	
Not sure	2 (1)	2 (1.1)	0	
Have heard about SMA				0.497
Yes	61 (30.8)	57 (31.5)	4 (23.5)	
No	137 (69.2)	124 (68.5)	13 (76.5)	

^a^Comparison between 2 groups, accepted vs. declined the screening test. CSMBS, Civil Servant Medical Benefit Scheme for government and state enterprise employee.

**FIGURE 2:**
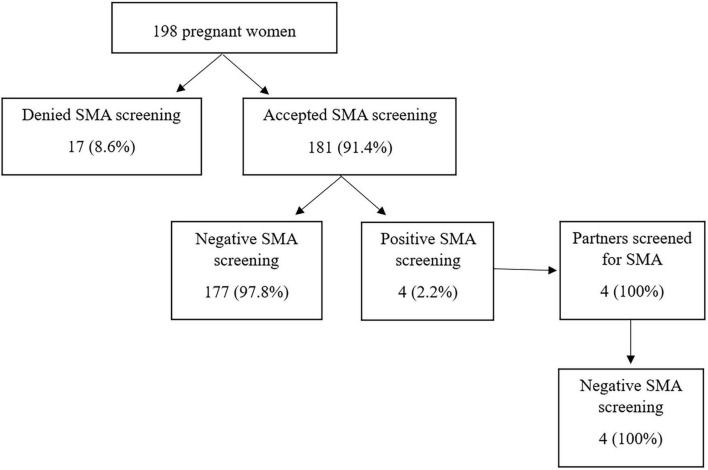
Prenatal carrier screening among 198 pregnant women.

Seventeen of 198 individuals declined the screening test while 181 took the test. The baseline characteristics of those who underwent screening and those who did not were compared, which showed no statistical difference (Table 1). Among those taking the test, four (4/181) were identified as SMA carriers; their partners were subsequently screened, which revealed non-carrier status, resulting in no couple at risk detected in the present study (Figure 2).

### Acceptance and attitudes toward SMA carrier screening

Around 95% or over of the participants agreed that SMA is a serious disorder resulting in severe life suffering and that the carrier screening could reduce unnecessary anxiety during pregnancy and should be offered to all pregnant women (Table 2). About 80%–92% or more of the participants expressed that having a child with SMA would complicate their life, they were not reluctant to have the screening test done, they had good faith in the accuracy of the test, and they would have a fetal diagnosis if they would be found to be a couple at risk (Table 2). Almost half of the participants expressed their tendency to choose termination of pregnancy for an affected fetus (Table 2).

**TABLE 2:** The participants’ attitudes toward the severity and burden of SMA and prenatal SMA carrier screening, after pre-test counseling (*N* = 198).

Questions	Agree %	Not sure %	Disagree %
SMA is a very severe genetic disease that leads to long-term suffering and difficulty living in society.	94.4	5.6	0
If I had to raise a child with SMA, it would cause me chronic burdens and complicated life.	83.8	0.5	15.7
Having prenatal SMA carrier screening can help reduce my anxiety related to the risk of having an affected child during pregnancy.	94.4	5.6	0
I am not hesitant to have a blood test for prenatal SMA carrier screening.	86.4	12.1	1.5
I have confidence that the SMA carrier screening has high accuracy.	79.3	20.7	0
If the screening test indicates that we (my partner and I) are at risk of having a fetus with SMA, we will get a fetal diagnosis.	92.4	6.1	1.5
If the result of fetal diagnosis indicates an affected fetus, we would decide to have a pregnancy terminated.	47	49	4
Prenatal counseling and SMA carrier screening should be offered to all pregnant women.	98	2	0
To get SMA carrier screening, I am willing to self-pay, although the cost may be high.^a^	48.5	45.4	6.1

^a^Based on the participants’ self-perception of “high cost,” the researchers did not provide specific number (exact price).

In the present study, the most common reasons for having the carrier screening included wanting to know their carrier status (87.8%), being worried about having an affected fetus (72.9%), and no extra cost for the test (50.3%) (Table 3). As for those declining the test, they reasoned that SMA was rarely found (58.8%) (Table 3).

**TABLE 3:** The reasons for choosing “to have” and “not to have” the SMA carrier screening test.

Reasons/rationale thinking of pregnant women (*N* = 198)	*N*	%
Those choosing “to have” the carrier screening (*N* = 181): ≥ 1 answer selected	(181)	(100)
I want to know my carrier status.	159	87.8
I am nervous that the fetus could have SMA.	132	72.9
I don’t have to pay an extra fee.	91	50.3
I want to provide my data to the researchers.	80	44.2
Others	1	0.6
Those choosing “not to have” the carrier screening (*N* = 17): ≥ 1 answer selected	(17)	(100)
I am not worried about SMA because it is rarely		
found.	10	58.8
I would not get a fetal diagnosis even though we		
(my husband and I) are found to be a couple at		
risk, so there is no point in doing the carrier		
screening.	1	5.9
I would not choose termination of pregnancy		
despite the affected fetus.	3	17.6
I don’t want to know my genetic defects (if there are		
any).	0	0
I don’t want to risk the relationship with my partner		
(if I am found to be a carrier).	0	0
I am afraid that going through the process of testing		
may cause me anxiety.	2	11.8
I don’t want to get pain from blood drawing.	3	17.6
My partner and/or relatives do not support me to		
have the test done.	1	5.9
Others	1	5.9

Almost half of the participants (47.7%) said that they could afford 501–1,000 Baht (∼14–28 USD) if they self-pay for the SMA carrier screening. Most participants (95.5%) thought that the cost of carrier testing should be covered by the government and/or their health coverage schemes (Table 4).

**TABLE 4:** Participant’s opinion on the affordability of the SMA carrier screening.

Participant’s opinion	*N*	%
The cost for SMA carrier screening that the participant can afford, if the participant has to self-pay (*N* = 195)	(195)	(100)
< 500 Baht	50	25.6
501–1,000 Baht	93	47.7
1,001–3,000 Baht	33	16.9
> 3,000 Baht	19	9.7
In the participant’s opinion, SMA carrier screening for pregnant women should be covered/paid by (*N* = 197)	(197)	(100)
The pregnant women themselves–self-pay	9	4.6
Healthcare coverage schemes (UHC, SSS,		
or CSMBS, according to their eligibility)	86	43.7
The government pays for all, regardless of the		
women’s type of healthcare coverage	102	51.8

CSMBS, Civil Servant Medical Benefit Scheme; SSS, Social Security Scheme; UHC, Thai universal health coverage scheme.

After receiving the result of the screening test, all the participants expressed a feeling of gladness that they had had the test done. They all agreed that the information about SMA provided to them was very useful and that post-test counseling was essential (Table 5).

**TABLE 5:** The participants’ attitudes toward the prenatal SMA carrier screening, after disclosure of the test result (*N* = 180).

Questions	Agree %	Not sure %	Disagree %
I feel glad that I had the test done.	100	0	0
The information about SMA provided to me is very much useful.	100	0	0
The post-test counseling is essential.	100	0	0
The whole process of the carrier screening is time-worthy.^a^	99.4	0	0.6
SMA carrier screening should be offered to all pregnant women.	98.3	1.7	0

^a^From pre-test counseling till having the test done and completion of post-test counseling.

### SMA carrier rate and frequencies of SMN2 copy number

Only 2.2% (4/181) of the participants were found to be carriers (Table 6). As for the *SMN2*, the majority of participants had 2 copies (58.5%), followed by 1 copies (34.3%), 0 copies (5.5%), and ≥ 3 copies (1.7%). Regarding the carrier individuals, three had 2 copies and the other had 1 copy of *SMN2*.

**TABLE 6:** Copy number of *SMN1*-exon 7 and *SMN2*-exon 7 in the participants (*N* = 181).

Gene	*SMN1*-exon 7		*SMN2*-exon 7	
	Copy number	Number of individuals	Copy number	Number of individuals
All participants			0	10
	1	4	1	62
	2	169	2	106
	3	8	3	2
			4	1
		181		181
Carrier only	1	4	1	1
			2	3
		4		4

## Discussion

We found a high acceptance rate for prenatal SMA carrier testing (91.4%) and positive attitudes toward the screening test, among the pregnant women in this study. Most participants (92%) would choose fetal diagnosis if they were found to be a couple at risk and considered having an interruption of pregnancy in case of the affected fetus (47%); these findings were in agreement with earlier studies (13, 15). The majority of the participants suggested SMA carrier testing to be part of standard antenatal care and covered by the public health system.

The low awareness of SMA among the participants was probably due to the rarity of the disorder; being a hidden problem of SMA owing to the short life of a significant proportion of the patients, the patients’ low social activities because of respiratory and ambulatory difficulties; and the lack of public health education and screening services for SMA (9, 28, 29).

The acceptance rate for SMA prenatal carrier screening was high among the participants in the present study, despite low awareness of SMA prior to the enrollment, this was likely due to several factors including the knowledge about SMA given, perceived usefulness of the pre-and post-test counseling provided, trust in the test offered, testing at no extra cost (Table 3), and availability of the downstream services. Some studies have shown a significantly increased acceptance rate if covered by medical insurance/health scheme (97.8%) compared to self-funded screening (81.1%) (29, 30). Other influencing factors included race, parity, and religion (17, 29).

As for those declining the screening test, the main reasons were the low prevalence of SMA (58.8%) and not choosing to interrupt the pregnancy for an affected fetus (17.6%). Our findings were consistent with previous reports that people without SMA experience had a significantly lower rate of taking carrier testing because they had a dimmer view of the condition than those with experiential knowledge (29, 31). Therefore, endorsement from public health authorities and professional society and effective mass communications are urgently needed in advancing the SMA carrier screening program (29–31).

The SMA carrier rate found in this study was 1 in 45, with an estimated prevalence of SMA at 1 in 8,264, similar to those found worldwide (11, 13, 15–21, 26, 27). The majority (98.3%) of the participants had ≤ 2 copies of *SMN2*, suggesting that a major proportion of Thai-affected individuals (fetuses) would be predicted to have type I SMA, consistent with other studies (2, 11). However, a small percentage (up to 5.5%) of the patients could be affected with SMA type 0, which is the most severe form, and the efficacy of currently available genetically designed therapies is still questionable.

Potentially, there could be an overwhelming emotional and psychological effect on the couple if both partners were found to be SMA carriers, as well as moral challenges related to making the decision about continuing or terminating a high-risk pregnancy. Therefore, the pretest and posttest counseling must be well prepared and provided, based on the principles of genetic counseling, namely respect for autonomy, non-judgemental, and confidentiality. Continuing support and necessary downstream actions, including discussion with other families previously or currently having a child with SMA, should be in place, as needed. Disease-modification therapies and their efficacy and limitations, including affordability, should be discussed with the couple.

To establish a new prenatal carrier screening program in any country, it is essential to have testing that is acceptable to the target population, with timely turnaround time, appropriate pre-test and post-test counseling, and affordable downstream services including testing for the partners, fetal diagnosis, and choices of continuation and interruption of pregnancy. Other local contexts, such as affordable treatment and predicted severity of the disorder, would also tremendously influence the individual’s decision on taking or declining the testing. Therefore, population-specific data, both biomedical (carrier rate, copy number of *SMN2*, type of SMA, availability of specific treatment) and sociocultural aspects (religion and extended socio-educational supports) are highly essential for policy planning for each country.

SMA fits all the criteria for prenatal carrier screening, as follows: (1) severity of the disorder; (2) high carrier frequency in the general population of more than 1 in 100; (3) available carrier screening test with a high sensitivity and specificity; (4) availability of prenatal diagnosis during early gestation; and (5) timely turnaround time for the screening test and fetal diagnosis (22, 28).

The impact of population-based carrier screening for SMA has been well shown in Israel by that 31% of the cases were prenatally diagnosed following the introduction of the screening through healthcare service and insurance plans (since 2008), and even higher at 52% after it became the national program (since 2013) (30).

The average monthly income per household of the participants of the present study was comparable to or a bit higher than the national average, 52%: 15,000–29,999 Thai Baht (THB) and 28%: 30,000–50,000 THB vs. 27,352 THB, according to the 2021 data from the National Statistical Office (Table 1).^2^ In the present study, the copy number analysis of both *SMN1* and *SMN2* (MLPA method) cost 3,000 THB (∼84 USD). However, if we offer the SMA carrier screening (MLPA method) to general pregnant women (negative family history), only *SMN1* will be offered, which costs about 1,500 THB (∼42 USD). The cost of screening tests is, therefore, about 5.5% of the average monthly household income. The cost of screening is a little higher than the willingness to self-pay or the affordability of the participants 73.3% were willing to pay 1,000 THB (28 USD) or lower (Table 4). This data supports that the economic intervention for the SMA screening test should be included in the policy preparation to reduce and prevent inequality in access to screening.

To date, Thailand has had a successful prenatal carrier screening program for thalassemia using a sequential testing strategy. Both thalassemia and SMA are inherited in an autosomal recessive fashion. Prenatal thalassemia carrier screening requires 6 ml of peripheral blood and is offered to all pregnant women (couples) at any stage of pregnancy despite preference at < 14-week gestational age. Prenatal SMA carrier screening could be performed at the same time as thalassemia screening without the need for additional blood collected if the genetic testing for both disorder are performed at the same laboratory/institute, otherwise, an additional 3–5 ml of blood in a separate tube from thalassemia is needed for *SMN* analysis.

We believe that it is highly pragmatic to incorporate the SMA screening along with the thalassemia screening program for the Thai population. However, public education about SMA and additional training for healthcare professionals, especially those in family medicine and prenatal settings are required. Moreover, the SMA carrier screening should be further expanded to the pre-conceptional period, when timing would allow broader reproductive choices without the time pressures imposed by pregnancy milestones.

To our knowledge, this study is the first study to evaluate the acceptance of prenatal SMA carrier screening and its influencing factors among the Thai population, using a prepared set of educational materials and counseling programs. The study is one of very few studies of its kind among Asian populations and low-middle-income countries which is underrepresented in the literature. The validated Thai-language brochure and the whole process of testing strategy, which were approved for their usefulness by the participants could be applied to a large-scale screening among the Thai population. Lastly, this is the first demonstration of the copy number of *SMN2* among the general population of Thais.

SMA newborn screening and disease-modification therapies (one-time gene therapy and lifelong splicing modification treatment) have been implemented in several high-income countries such as Taiwan, the US, and European countries (32–35). The main purpose of the newborn screening is to detect cases early, which allows the opportunity for the infant to receive effective treatment if given in the presymptomatic period (35, 36). Unfortunately, given that these new therapeutics are extremely expensive and not reimbursable in many countries, including Thailand, it could create a conflicting scenario of newborn screening without treatment availability in such countries. On the other hand, the main purpose of prenatal/preconceptional screening is for prevention in most cases. Moreover, there are pros and cons from the perspective of ethical dilemma as well as health economics of the newborn screening vs. the prenatal/preconceptional carrier screening. While the disease-modification therapies are shown to be effective in early childhood, it is important for physicians to discuss with families the fact that their long-term outcome (> 10 years) and risk-benefit ratio remain unknown (35, 36). In a recent study in a country where SMA newborn screening is available and the parents value early diagnosis and treatment, the majority of them preferred pre-conception and/or prenatal screening to newborn screening (37). While SMA newborn screening and reimbursable treatment are still struggling in Thailand, we would like to provide an alternative for primary prevention to the public at large.

The authors appreciated the limitations of the study, including the small sample size and the absence of couples at risk and affected fetuses identified, which might have resulted in different answers on choosing fetal diagnosis and pregnancy outcome. There were also 4 eligible participants who declined to participate in the study, which could lead to a bias in the selection of the eligible sample. This number accounts for a minor fraction, 2.0% (4/202); therefore, we believe that this is less likely to interfere with the main findings of the present study, though a small effect is possible. To avoid the potential feeling of discomfort from being asked and ethical concerns, we did not probe their reasons.

## Conclusion

We found a positive attitude and a high acceptance rate for prenatal SMA carrier screening among pregnant Thai women, and it could be further amplified by effective mass education to the general population and affordable screening services. The estimated prevalence and carrier frequency of SMA among the Thai population were found to be equivalent to the worldwide data, and the majority of patients were predicted to have the most severe type of SMA, necessitating the need for a national program for disease prevention. Population-specific data, both biomedical and sociocultural aspects, are markedly important for national policy planning for carrier screening.

## Data Availability

The original contributions presented in this study are included in this article/Supplementary material, further inquiries can be directed to the corresponding author.

## Footnotes

1
https://eng.nhso.go.th/

2
https://www.nso.go.th/public/e-book/statistical-yearbook/syb-2022/58
