# Supplementary material for: Prenatal carrier screening for spinal muscular atrophy among pregnant Thai women
Source: Front Med (Lausanne). 2025 Jun 23;12:1566417. doi: 10.3389/fmed.2025.1566417 (PMC12229863; doi:10.3389/fmed.2025.1566417)

โรคเอสเอ็มเอ

คือ โรคกล้ามเนื้ออ่อนแรงแต่กำเนิด เกิดจากการเสื่อมลงของเซลล์ประสาทสั่งการที่อยู่ในไขสันหลัง เซลล์ประสาทสั่งการจึงไม่สามารถส่งสัญญาณไปยังกล้ามเนื้อให้ทำงานตามปกติได้ ส่งผลให้กล้ามเนื้อฝ่อและอ่อนแรงลงอย่างต่อเนื่อง โรคนี้คาดว่าพบได้ประมาณ 1 ใน 10,000 ของประชากร

อาการของโรคเอสเอ็มเอ

ผู้ป่วยจะเริ่มมีอาการได้ตั้งแต่แรกเกิดจนถึงวัยผู้ใหญ่ขึ้นกับชนิดของโรค อาการแสดงในวัยเด็กได้แก่ ตัวอ่อน ไม่สามารถชันคอ นั่งหรือยืนได้เอง ดูดและกลืนลำบาก โตช้า หายใจลำบาก สำลักง่ายและติดเชื้อทางเดินหายใจส่วนล่างบ่อยครั้ง อาการแสดงในวัยผู้ใหญ่ได้แก่ กล้ามเนื้อต้นแขนและขาอ่อนแรงทั้งสองข้างซึ่งเป็นมากขึ้นเรื่อยๆ

ชนิดของโรคเอสเอ็มเอ

มี 4 ชนิด แบ่งตามอายุที่เริ่มแสดงอาการและความรุนแรงของโรค

ชนิดที่	อายุที่เริ่มแสดงอาการ	ความรุนแรง	อายุขัยเฉลี่ย
1	ก่อน 6 เดือน	มากที่สุด	8 - 10 เดือน
2	6 - 18 เดือน	มาก	25 ปี
3	หลัง 18 เดือน	ปานกลาง	ปกติ
4	20 - 30 ปี	ปานกลาง	ปกติ

ชนิดที่ 1 เป็นชนิดที่พบได้บ่อยที่สุด ผู้ป่วยจะเริ่มมีอาการภายในอายุ 6 เดือน กล้ามเนื้ออ่อนแรงรุนแรง ระบบทางเดินหายใจล้มเหลวและมักเสียชีวิตภายในปีแรกหลังเกิด

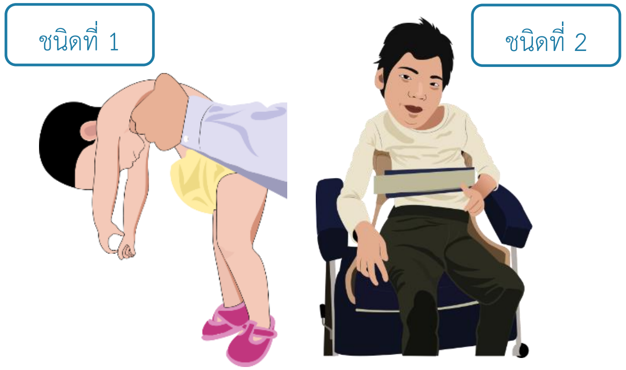

ชนิดที่ 2 เริ่มมีอาการตั้งแต่อายุ 6 เดือนถึง 1 ปีครึ่ง กล้ามเนื้ออ่อนแรงมากขึ้นเรื่อยๆ ลุกนั่งลำบาก หลังคด หายใจลำบาก ต้องใช้เครื่องช่วยพยุงให้นั่งได้และอาจต้องใช้เครื่องช่วยหายใจร่วมด้วย

ชนิดที่ 3 และ 4 อาการมีความหลากหลายและรุนแรงน้อยกว่า 2 ชนิดแรก อาการเป็นมากขึ้นเรื่อยๆจนไม่สามารถลุกขึ้นเองได้ในช่วงอายุ 20-50 ปี

ในระยะยาวผู้ป่วยที่ไม่สามารถนั่งหรือเดินได้ จะประสบปัญหาหลังคด ข้อยึดติด และปอดขยายตัวไม่ดีทำให้หายใจลำบากจนต้องใช้เครื่องช่วยหายใจ

สาเหตุของโรคเอสเอ็มเอ

เกิดจากการกลายพันธุ์ของยีน *SMN1* ทำให้ไม่สามารถสร้างโปรตีนที่จำเป็น สำหรับการทำงานของเซลล์ประสาทสั่งการได้

การรักษา

ทำได้โดยการรักษาแบบประคับประคองตามอาการ แม้ว่าปัจจุบันมีวิธีการรักษาให้หายได้ด้วยยาและยีนบำบัดแต่ค่าใช้จ่ายในการรักษาต่อรายสูงถึงหลายสิบล้านบาทต่อปีรวมถึงผลการรักษายังมีความหลากหลายในผู้ป่วยแต่ละราย

โอกาสเสี่ยงของการมีลูกเป็นโรคเอสเอ็มเอ

โรคเอสเอ็มเอเป็นโรคที่ถ่ายทอดทางพันธุกรรมแบบยีนด้อย โดยผู้ที่มียีนด้อยแฝงอยู่จะเรียกว่าเป็นพาหะ ซึ่งจะไม่แสดงอาการของโรค แต่ถ้าคู่สมรสเป็นพาหะด้วยกันทั้งคู่จะสามารถถ่ายทอดยีนด้อยนี้ไปให้แก่ลูก และมีโอกาสทำให้ลูกเป็นโรคเอสเอ็มเอได้

การศึกษาในประชากรไทยทั่วไปพบว่าในประชากร 50 รายจะมีคนเป็นพาหะ 1 ราย ซึ่งคาดว่าน่าจะสูงเป็นอันดับสองรองจากโรคธาลัสซีเมีย ดังนั้นผู้ที่เป็นพาหะมีโอกาสจะมีลูกเป็นโรคได้แม้ว่าจะไม่เคยมีประวัติคนในครอบครัวเป็นโรคเอสเอ็มเอก็ตาม

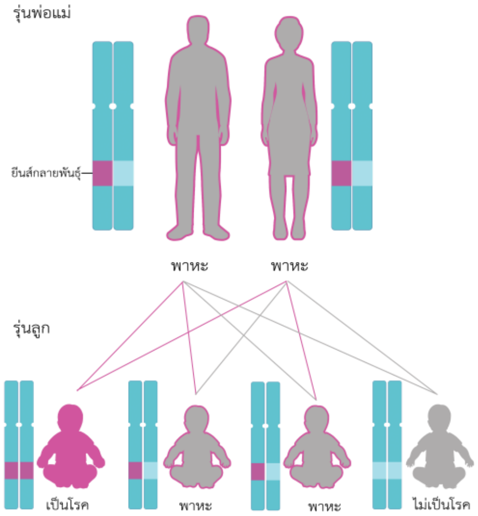

คำแนะนำสำหรับผู้ที่เป็นพาหะโรคเอสเอ็มเอ

ผู้ที่เป็นพาหะจะไม่มีอาการผิดปกติแต่อย่างใด แต่ถ้าตรวจพบว่าทั้งหญิงตั้งครรภ์และคู่สมรสเป็นพาหะ ความเสี่ยงที่จะมีลูกเป็นโรคเอสเอ็มเอคือ 1 ใน 4 ของทุกการตั้งครรภ์

การตรวจคัดกรองพาหะโรคเอสเอ็มเอ

สามารถทำได้โดยการเจาะเลือดหญิงตั้งครรภ์เพื่อตรวจว่าเป็นพาหะของโรคเอสเอ็มเอหรือไม่ ซึ่งปัจจุบันพบว่าการตรวจมีความไวร้อยละ 95 หญิงตั้งครรภ์ที่ตรวจพบว่าตนเองเป็นพาหะควรให้คู่สมรสมาตรวจเลือดคัดกรองพาหะโดยเร็วที่สุด

การตรวจวินิจฉัยทารกในครรภ์

สามารถทำได้หลายวิธี ได้แก่ การเจาะชิ้นเนื้อรก การเจาะน้ำคร่ำและการเจาะเลือดสายสะดือทารก ทั้งนี้ขึ้นอยู่กับอายุครรภ์ อย่างไรก็ตามควรตรวจวินิจฉัยภายในอายุครรภ์ 19 - 21 สัปดาห์

คำแนะนำสำหรับผู้ที่ตรวจพบว่าทารกในครรภ์เป็นโรคเอสเอ็มเอ

หญิงตั้งครรภ์และคู่สมรสควรได้รับคำปรึกษาจากทีมแพทย์ผู้เชี่ยวชาญโดยเร็วที่สุด เพื่อพิจารณาร่วมกันถึงทางเลือกในการตรวจติดตามทารกหลังเกิดและรักษาตามอาการ หรือทางเลือกในการยุติการตั้งครรภ์ก่อน 24 สัปดาห์ ตามข้อบ่งชี้ทางการแพทย์

ตรวจเลือดเพื่อคัดกรองพาหะ "โรคเอสเอ็มเอ"

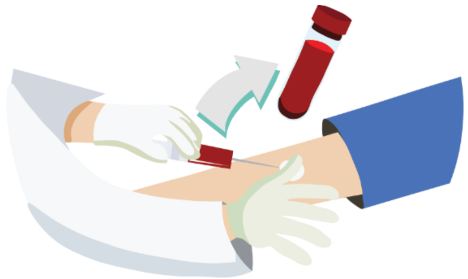

ถ้าตรวจพบว่าเป็นคู่เสี่ยง
สามารถตรวจวินิจฉัยทารกในครรภ์ได้

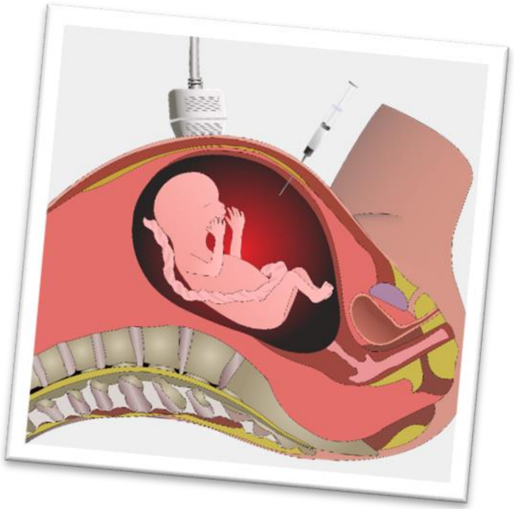

เพื่อเตรียมความพร้อมหรือหลีกเลี่ยง
การมีลูกเป็นโรคเอสเอ็มเอ

สอบถามข้อมูลเพิ่มเติมติดต่อ
อ.พญ.ชยดา ตั้งชีวินศิริกุล หรือ คุณมณีรัตน์ ประกอบพาณิชย์
เบอร์โทรศัพท์ 02-2012166 หรือ 089-8905444 หรือ 081-8994626

โครงการตรวจคัดกรองพาหะโรคเอสเอ็มเอในหญิงไทยที่ตั้งครรภ์
โรงพยาบาลรามาธิบดี มหาวิทยาลัยมหิดล

โรคเอสเอ็มเอ

Spinal Muscular Atrophy
(โรคกล้ามเนื้ออ่อนแรงแต่กำเนิด)

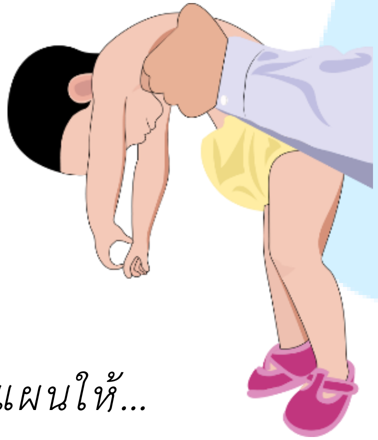

ตรวจก่อนให้รู้
เพื่อวางแผนให้...
ครอบครัวที่ "คุณรัก"

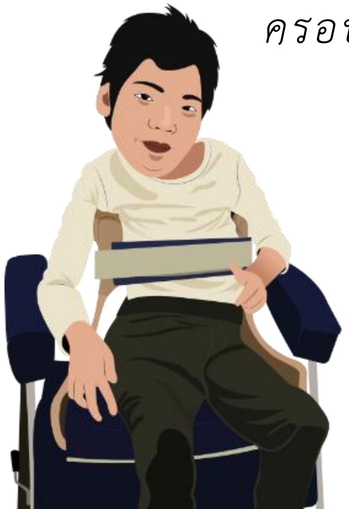

Supplement: Supplementary file 1 [file Data_Sheet_1.pdf]
