# Supplementary material for: Prenatal carrier screening for spinal muscular atrophy among pregnant Thai women
Source: Front Med (Lausanne). 2025 Jun 23;12:1566417. doi: 10.3389/fmed.2025.1566417 (PMC12229863; doi:10.3389/fmed.2025.1566417)
Supplement: Supplementary file 2 [file Data_Sheet_2.pdf]

Spinal Muscular Atrophy (SMA)

SMA is a genetic neuromuscular disease caused by the degeneration of motor neurons in the spinal cord of the patient. The affected motor neurons cannot transmit signals to the muscles to function normally, which results in gradual and progressive muscle weakness. The prevalence of this disorder is expected to be approximately 1 in every 10,000 people.

Symptoms of SMA

The symptoms of SMA may develop at any time from birth through adulthood, depending on the type of SMA. The symptoms in infancy and childhood include infantile floppiness, not being able to hold their head up, not being able to sit or stand, difficulty sucking and swallowing, failure to gain weight and grow normally difficulty breathing, choking easily, and recurrent infection of the lower respiratory tract. For those who develop symptoms later in adulthood, the symptoms include muscle weakness of the upper arms and legs, which could be worsening over time.

Types of SMA

There are 4 types of SMA, based on their severity and the age at which symptoms start.

Type	Age at symptoms start	Severity	Average lifespan
1	Before 6 months	Most severe	8–10 months
2	6 – 18 months	Severe	25 years
3	After 18 months	Moderate	Normal
4	20-30 years	Moderate	Normal

Type 1 is the most common type. The symptoms are most severe and begin before 6 months of age, which often leads to respiratory failure and death during infancy unless vigorous and ventilatory support is provided.

(Image) (Image)

Type 2 leads to the symptoms occurring during 6 months and 1.5 years. There is slowly progressive muscle weakness, difficulty standing up from a sitting position, progressive scoliosis, and difficulty breathing. Later, the patients may require sitting support and/or ventilator support.

Types 3 and 4 are less severe compared to the first 2 types and they are associated with varying degrees of muscle weakness. However, it could lead to non-ambulation by the age of 20-50 years in some patients.

In the long term, non-ambulatory patients are likely to have scoliosis, joint contracture, and impaired lung expansion (restrictive lung disease) which may cause respiratory failure and require ventilator support.

Causes of SMA

SMA is caused by genetic defects of the *SMN1* gene, which result in impaired production of a protein that is essential for motor neuron functions.

Treatments

Treatments for SMA include symptomatic and supportive management. Specific treatments for SMA are recently available, which are medicinal treatment and gene therapy. However, these new treatments are costly, over 10 million Baht per year, and the outcomes of treatment may vary from case to case.

The risk of having a child with SMA

SMA is inherited in autosomal recessive mode. A carrier individual is asymptomatic. If a couple are both carriers, then they have a chance of passing on the genetic defect to their child. The child who inherits the defective gene from both parents will be affected by SMA.

Studies from the Thai general population indicate a carrier frequency of 1 in every 50 people, which is prevalent and second to only thalassemia. Couples with SMA carriers have a high chance of having a child with SMA even though they have a negative family history.

[Graphic of inheritance pattern]

Advice for individuals who are carriers of SMA

A carrier individual is asymptomatic, meaning that carrier status does not affect the individual's health. If a pregnant woman and her partner are both found to be SMA carriers, the couple has a <u>1 in 4</u> chance of having a child with SMA for each pregnancy.

Prenatal carrier screening for SMA

A pregnant woman can choose to have a blood test for SMA carrier screening. The sensitivity of the screening test is about 95 percent. The pregnant woman who is found to be an SMA carrier is suggested to have her partner to be tested for SMA carrier status as soon as possible.

Fetal diagnosis

There are several methods to detect if the fetus is affected by SMA or not, such as chorionic villus sampling, amniocentesis, and fetal blood sampling, depending on the stage of pregnancy. However, the fetal diagnosis should be performed no later than 19-21 weeks of gestation.

Advice for those found to have an affected fetus with SMA

The couple is advised to get post-test counseling as soon as possible, including information from specialists in SMA. Options should be offered to the couple, including delivery of the affected child followed by available and affordable treatments and interruption of pregnancy, which is legally allowed within 24 weeks of gestation.

Blood test for the prenatal SMA carrier screening

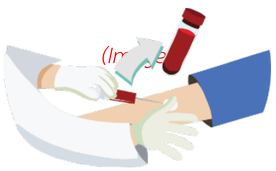

For couples at risk,
prenatal or fetal diagnosis is a possible option

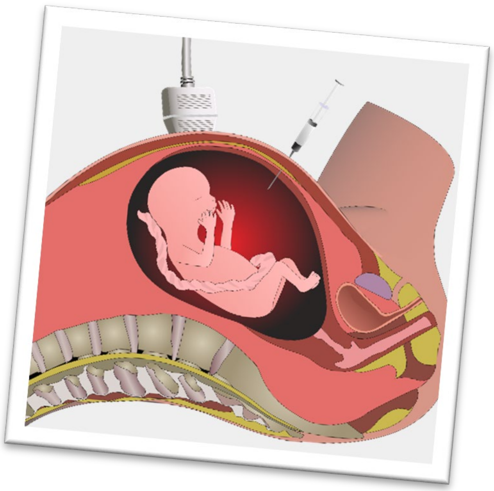

(Image)

This is for preparation for the birth and following treatments or to avoid having a child with SMA

For more information, please contact:
Dr. Chayada Tangshewinsirikul or Ms.Maneerat Prakobpanich
Phone: 02-2012166 or 089-8905444 or 081-8994626

Project

Prenatal Carrier Screening for Spinal Muscular Atrophy (SMA) among Thai Pregnant Women
Ramathibodi Hospital, Mahidol Univer:

Hospital

Logo

SMA

Spinal Muscular Atrophy

(Congenital muscular weakness)

(Image)

Test it now to know

and plan for ...

your ***"beloved"*** family

(Image)
