# Supplementary material for: Prenatal carrier screening for spinal muscular atrophy among pregnant Thai women
Source: Front Med (Lausanne). 2025 Jun 23;12:1566417. doi: 10.3389/fmed.2025.1566417 (PMC12229863; doi:10.3389/fmed.2025.1566417)
Supplement: Supplementary file 3 [file Data_Sheet_3.docx]

**Questionnaire for Research**

No.__________

**Prenatal Carrier Screening for Spinal Muscular Atrophy (SMA)**

**among Thai pregnant women**

**Explanation:**

1. The purpose of this questionnaire is to collect information to assess attitudes toward SMA and prenatal carrier screening for SMA in pregnant Thai women.
2. The information in this questionnaire will only be used to benefit research. The research participants have the right to quit the research participation at any time without providing the quitting reason, and it will not affect the medical treatment or other rights in the future.
3. This questionnaire consists of 3 parts, namely:

Part 1: General Information

Part 2: Attitudes toward the severity and burden of SMA and its impact on the decision for prenatal SMA

carrier screening

(Participants provide answers after receiving counseling about SMA and SMA prenatal carrier

screening

Part 3: Opinion on receiving the prenatal carrier screening for SMA.

Part 4: Attitudes and experiences toward the prenatal SMA carrier screening

(Participants provide answers after disclosure of the result of carrier testing)

………………………………………………………..

**Part 1: General Information**

**Explanation:** Please mark ✓ in the box you wish to answer and fill in the blank with text or numbers.

1. Age ………....… Years (years only)
2. Place of current residence

☐ Bangkok ☐ Other province (please specify) ……………………………….

1. Religion

☐ Buddhist ☐ Christian ☐ Muslim ☐ Other (please specify) …………………

☐ Prefer not to say

1. Highest education

☐ Less than a primary school diploma ☐ Primary school diploma

☐ High school diploma/ Vocational certificate ☐ High Vocational certificate Associate’s degree

☐ Bachelor’s degree ☐ Master’s degree or equivalent ☐ Ph.D. or equivalent

1. Occupation

☐ Company employee / Private sector employee ☐ Causal worker/ Day laborer

☐ Housewife / Unemployed ☐ Personal business/ Self-employed

☐ Government officer/employee or State enterprise employee

☐ Healthcare professional (Please specify) ……………………………. (physician, dentist, physician or dentist assistant, nurse or associates, pharmacist or associate, medical technologist, physiotherapist, radiologic technologist, psychologist, public health officer)

☐ Other (Please specify) …………………………….

1. Family’s average monthly income

☐ Below 15,000 Baht ☐ 15,000 – 29,999 Baht

☐ 30,000 – 50,000 Baht ☐ Over 50,000 Baht

1. Type of healthcare coverage

☐ Universal Health Coverage (UHC or 30 Baht card/ Medical golden card)

☐ Social Security Scheme (SSS)

☐ Civil Servant Medical Benefit Scheme (CSMBS: Government officer/employee or State enterprise)

☐ Company welfare ☐ Private Health Insurance

☐ Cash ☐ Other (Please specify) …………………………….

1. Marital status

☐ Single ☐ Married ☐ Widowed ☐ Divorced/Separated

☐ Prefer not to say

1. Method of conception for this pregnancy

☐ Natural conception

☐ Using Assisted Reproductive Technology, e.g.Ovarian Stimulation, In-Vitro Fertilization (IVF)

1. Altogether, this pregnancy is your …………… time.
2. How many children have you given birth to? ……………….(child/children)
3. What is the number of miscarriages you have had? ……………….. time(s).
4. Have you ever had a child with a genetic condition or congenital disability/anomalies?

☐ Yes (please specify) …………………………………….…........................

☐ No ☐ Not sure

1. Have you ever had a relative with a genetic condition or congenital disability/anomalies?

☐ Yes (please specify) …………………………………….…........................

What is this person’s relationship to you? ……………….................................…………

☐ No ☐ Not sure

1. Do you have any chronic diseases?

☐ Yes (please specify) …………………………………….…........................

☐ No ☐ Not sure/ Don’t know

1. Does anyone accompany you for the antenatal care today?

☐ No, I come alone ☐ Yes, my partner ☐ Yes, my relative

☐ Yes, other (please specify) …………………………………….….

1. Have you ever heard about SMA?

☐ Yes, from which source of information? (You can choose more than one answer)

☐ my doctor or other healthcare professionals ☐ Exhibition/ Conference/ Billboard/ Poster

☐ Brochure/ Flyer ☐ Newspaper ☐ Internet / social media

☐ Television ☐ Radio ☐ Other (please specify) ………………………

☐ No

**Part 2 Attitudes toward the severity and burden of SMA and its impact on the decision for prenatal SMA carrier screening**

(Please provide answers after you received counseling about SMA and SMA prenatal carrier screening)

Explanation: Please mark ✓ in the box that matches your answer.

1. SMA is a very severe genetic disease that leads to long-term suffering and difficulty living in society.

☐ agree ☐ not sure ☐ disagree

1. If I have to raise a child with SMA, it would cause me chronic burdens and complicated life.

☐ agree ☐ not sure ☐ disagree

1. Having prenatal SMA carrier screening can help reduce my anxiety related to the risk of having an affected child during pregnancy

☐ agree ☐ not sure ☐ disagree

1. I am not hesitant to have a blood test for prenatal SMA carrier screening.

☐ agree ☐ not sure ☐ disagree

1. I have confidence that the SMA carrier screening has high accuracy.

☐ agree ☐ not sure ☐ disagree

1. If the screening test indicates that we (my partner and I) are at risk of having a fetus with SMA, we will get a fetal diagnosis.

☐ agree ☐ not sure ☐ disagree

1. If the result of fetal diagnosis indicates an affected fetus, we would decide to have a pregnancy terminated

☐ agree ☐ not sure ☐ disagree

1. Prenatal counseling and SMA carrier screening should be offered to all pregnant women.

☐ agree ☐ not sure ☐ disagree

1. To get SMA carrier screening, I am willing to self-pay, although the cost may be high

☐ agree ☐ not sure ☐ disagree

**Part 3 Opinions on receiving the prenatal carrier screening for SMA**

Explanation: Please mark ✓ in the box that matches your answer.

1. Which of the following is the reason for your decision *to have a blood test for* the prenatal SMA carrier screening? (You can choose more than one answer)

(In case you decided not to have the blood test, please skip to Question #2.)

☐ I want to know my carrier status

☐ I am nervous that the fetus could have SMA.

☐ I don’t have to pay an extra fee.

☐ I want to provide my data to the researchers.

☐ Other (please specify) …………………………………….….

1. Which of the following is the reason for your decision *not to have a blood test for* the SMA prenatal carrier screening? (You can choose more than one answer)

☐ I am not worried about SMA because it is rarely found.

☐ I would not get a fetal diagnosis even though we (my husband and I) are found

to be a couple at risk, so there is no point in doing the carrier screening.

☐ I would not choose termination of pregnancy despite the affected fetus

☐ I don’t want to know my genetic defects (if there are any).

☐ I don’t want to risk the relationship with my partner (if I am found to be a carrier).

☐ I am afraid that going through the process of testing may cause me anxiety.

☐ I don’t want to get pain from blood drawing.

☐ My partner and/or relatives do not support me to have the test done.

☐ Other (please specify) …………………………………….….

1. Do you agree that you have a chance of being an SMA carrier despite having no family history of SMA?

☐ I agree ☐ I am not sure ☐ I disagree

1. The cost for SMA carrier screening that you can afford, if you have to self-pay- is

☐ Below 500 Baht ☐ 501 – 1,000 Baht ☐ 1,001 – 3,000 Baht

☐ 3,001 – 5,000 Baht ☐ 5,001 – 7,000 Baht ☐ 7,001 – 9,000 Baht

☐ 9,001 – 10,000 Baht

1. In your opinion, SMA carrier screening for pregnant women should be covered/paid by whom?

☐ The pregnant women themselves or self-pay

☐ According to individual healthcare coverage schemes (UHC, SSS, or CSMBS)

☐ The government pays for all, regardless of the women’s type of healthcare coverage

**Part 4 Attitudes and experiences toward the prenatal SMA carrier screening**

(Please provide answers after you are informed about the result of carrier testing)

Explanation: Please mark ✓ in the box that matches your answer.

1. I feel glad that I had the test done.

☐ agree ☐ not sure ☐ disagree

1. The information about SMA provided to me is very much useful.

☐ agree ☐ not sure ☐ disagree

1. The post-test counseling is essential.

☐ agree ☐ not sure ☐ disagree

1. The whole process of the carrier screening is time-worthy (i.e. from pre-test counseling, blood test, waiting, for the result, testing partner, and post-test counseling)

☐ agree ☐ not sure ☐ disagree

1. SMA carrier screening should be offered to all pregnant women.

☐ agree ☐ not sure ☐ disagree

**Thank you** **for answering this questionnaire**
