# Supplementary material for: Prenatal carrier screening for spinal muscular atrophy among pregnant Thai women
Source: Front Med (Lausanne). 2025 Jun 23;12:1566417. doi: 10.3389/fmed.2025.1566417 (PMC12229863; doi:10.3389/fmed.2025.1566417)
Supplement: Supplementary file 4 [file Data_Sheet_4.docx]

**Supporting information S4 Table**

**Supplemental Table** Extended demographic data of participants

| Characteristic | Total  N = 198 (%) | Accepted screening test  N = 181 (%) | Declined screening test  N = 17 (%) |
| --- | --- | --- | --- |
| Maternal age (Years ± SD) | 32 ± 5.1 | 31.9 ± 5.2 | 33.6 ± 5 |
| Place of current residence  Bangkok  Another province | 152 (76.8)  46 (23.2) | 139 (76.8)  42 (23.2) | 13 (76.5)  4 (23.5) |
| Religion  Buddhist  Christian  Muslim  Other | 176 (88.9)  5 (2.5)  17 (8.6)  0 | 162 (89.5)  5 (2.8)  14 (7.7)  0 | 14 (82.4)  0  3 (17.6)  0 |
| Highest education  Less than a primary school diploma  Primary school diploma  High school diploma/ Vocational certificate  High Vocational certificateฝ Associate’s degree  Bachelor’s degree  Master’s degree or equivalent  Ph.D. or equivalent | 1 (0.5)  3 (1.5)  45 (22.7)  18 (9.1)  113 (57.1)  16 (8.1)  2 (1) | 1 (0.6)  3 (1.7)  39 (21.5)  16 (8.8)  105 (58)  15 (8.3)  2 (1.1) | 0  0  6 (35.3)  2 (11.8)  8 (47.1)  1 (5.9)  0 |
| Occupation  Company employee / Private sector employee  Causal worker/ Day laborer  Housewife / Unemployed  Personal business / Self-employed  Government officer/employee or State enterprise  employee  Healthcare professional  Other | 68 (34.3)  6 (3)  22 (11.1)  21 (10.6)  38 (19.2)    30 (15.2)  13 (6.6) | 63 (34.8)  5 (2.8)  21 (11.6)  16 (8.8)  37 (20.4)  27 (14.9)  12 (6.6) | 5 (29.4)  1 (5.9)  1 (5.9)  5 (29.4)  1 (5.9)  3 (17.6)  1 (5.9) |
| Family’s average monthly income  Below 15,000 Baht  15,000 – 29,999 Baht  30,000 – 50,000 Baht  Over 50,000 Baht | 19 (9.6)  104 (52.5)  56 (28.3)  19 (9.6) | 17 (9.4)  94 (51.9)  53 (29.3)  17 (9.4) | 2 (11.8)  10 (58.8)  3 (17.6)  2 (11.8) |
| Type of healthcare coverage (N = 197)  Universal Health Coverage (UHC)  Social Security Scheme (SSS)  CSMBS  Company welfare  Private health insurance  Cash  Other | 8 (4.1)  81 (40.9)  47 (23.7)  0  0  62 (31.3)  0 | 7 (3.9)  74 (40.9)  41 (22.7)  0  0  59 (32.6)  0 | 1 (5.9)  7 (41.2)  6 (35.3)  0  0  3 (17.6)  0 |
| Marital status  Single  Married  Widowed  Divorced/Separated  Prefer not to say | 33 (16.7)  164 (82.8)  0  0  1 (0.5) | 32 (17.7)  148 (81.8)  0  0  1 (0.6) | 1 (5.9)  16 (94.1)  0  0  0 |
| Method of conception  Natural conception  Using Assisted Reproductive Technology | 196 (99)  2 (1) | 180 (99.4)  1 (0.6) | 16 (94.1)  1 (5.9) |
| Gravida  Primigravida  Multigravida | 72 (36.4)  126 (63.6) | 69 (38.1)  112 (61.9) | 3 (17.6)  14 (82.4) |
| Parity  Nulliparity  Multiparity | 92 (46.5)  106 (53.5) | 86 (47.5)  95 (52.5) | 6 (35.3)  11 (64.7) |
| History of miscarriage  Yes  No | 137 (69.2)  61 (30.8) | 127 (70.2)  54 (29.8) | 10 (58.8)  7 (41.2) |
| Have had a child with a genetic condition or congenital disability/anomalies  Yes  No  Not sure | 8 (4)  188 (94.9)  2 (1) | 7 (3.9)  172 (95)  2 (1.1) | 1 (5.9)  16 (94.1)  0 |
| Have had a relative with a genetic condition or congenital disability/anomalies  Yes  No  Not sure | 6 (3)  182 (91.9)  10 (5.1) | 6 (3.3)  165 (91.2)  10 (5.5) | 0  17 (100)  0 |
| Have any chronic diseases  Yes  No  Not sure/ Don’t know | 41 (20.7)  157 (79.3)  0 | 38 (21)  143 (79)  0 | 3 (17.6)  14 (82.4)  0 |
| A person who accompanied the participant to the antenatal care today  None  Yes, partner  Yes, relative  Other | 77 (38.9)  118 (59.6)  3 (1.5)  0 | 69 (38.1)  109 (60.2)  3 (1.7)  0 | 8 (47.1)  9 (52.9)  0  0 |
| Have heard about SMA  Yes  No | 61 (30.8)  137 (69.2) | 57 (31.5)  124 (68.5) | 4 (23.5)  13 (76.5) |
